# Recent updates on the possible reasons for the low incidence and morbidity of COVID-19 cases in Africa

**DOI:** 10.1186/s42269-021-00589-9

**Published:** 2021-07-23

**Authors:** Emmanuel Kagning Tsinda, Gideon Sadikiel Mmbando

**Affiliations:** 1 Graduate School of Medicine, Tohoku University, Sendai, 980-8575 Japan; 2 Graduate School of Life Sciences, Tohoku University, Sendai, 980-8577 Japan

**Keywords:** COVID-19, SARS-CoV-2, Burden, Africa, Susceptibility, Severity

## Abstract

**Background:**

The COVID-19 respiratory illness caused by the SARS-CoV-2 has been a major cause of morbidity and mortality worldwide since the first reported case in Wuhan, China. A year has passed since pandemic began, and the reasons for different COVID-19 burden variation across continents keep puzzling the general public.

**Main body of the abstract:**

Since the COVID-19 pandemic started, published research articles have addressed the epidemiological risk factors, host factors, susceptibility and immunity. To ascertain possible reasons for the different rates of COVID-19 infections between Africa and other continents, we summarized the up-to-date scientific literature to identify possible arguments in this regard. Available literature suggests that demographic, epidemiological, sociological, genetic and immunological factors contribute in the COVID-19 severity and the susceptibly to SARS-CoV-2.

**Short conclusion:**

This review summarizes existing data and discusses reasons for differential COVID-19 burden across continents. The arguments mentioned herein will be helpful to guide future experimental studies to test different hypotheses.

## Background

As of March 2021, the severe acute respiratory syndrome coronavirus-2 (SARS CoV-2) has infected over 114,000,000 persons in 219 territories and caused more than 2,500,000 deaths globally (World Health Organization 2021a, b; Worldometers 2021). At the early stage of the SARS-CoV-2 outbreak in China and Europe, public health authorities all over the world had expressed concerns over the damage COVID-19 would cause when SARS-CoV-2 hit African countries (Nordling 2020a). SARS-CoV-2 was introduced in Africa mostly from Europe (Loembe’ et al. 2020), the incidence rate and severity of COVID-19 in African countries remains lower than expected (Nordling 2020b) and many are still wondering why Africa has not been hardly hit by the deadly COVID-19 pandemic. Previous attempts to explain the mysteriously low COVID-19 mortality and morbidity in Africa highlight the possible role of population demographics, distribution of COVID-19 risk factors, the climate, urbanization, economic level, the low testing rate and human immunity (Chitungo et al. 2020; Diop et al. 2020; Margolin et al. 2020; Mbow et al. 2020; Njenga et al. 2020; Nordling 2020b, c; Gesesew et al. 2021; Lawal 2021). Understanding the reasons for different incidence and morbidity of COVID-19 across regions and continents might reveal preventive strategies to guide public health interventions. Here, we review the literature and rely on updated scientific literature and social events in Africa to discuss additional factors that may or may not explain the low observed burden of COVID-19 in Africa.

## Main text

### Previously mentioned hypotheses for low COVID-19 incidence in some parts of the world

Several authors have expressed hypotheses on the reasons of the low prevalence and severity of COVID-19 infection in some countries. Iwasaki and Grubaugh suggested the likely reasons for the low infection rate in Japan. They argued that Japanese culture was suited for social distancing. They also argued that Japanese people might have been exposed to less pathogenic virus strains of the SARS-CoV-2 (Iwasaki and Grubaugh 2020). Authors of that paper also postulated that low ACE2 lung expression and distinct HLA of the Japanese might confer SARS CoV-2 resistance, but more data are needed to validate that hypothesis. The last argument in the paper of Professor Iwasaki is the possible cross-reactive protection conferred by the particular BCG vaccination implemented in countries such as Japan, Brazil and Russia. It seems possible that BCG vaccination deployment policy is correlated with a decreased number of COVID-19 deaths (Escobar et al. 2020), but a population-level case–control study in Israel did not find a significant association between BCG vaccination and protection of young adults against COVID-19 (Hamiel et al. 2020). A randomized controlled trial evaluating the efficacy of vaccination with BCG in the prevention of COVID-19 will help clarify the role of BCG in COVID-19 infection (Junqueira-Kipnis et al. 2020).

In Asian countries, the culture of wearing a mask is widely adopted by the population, as a result of the occurrence of influenza or SARS outbreaks in the past. But, the culture of wearing a mask was introduced into African culture just recently, due to the COVID-19 pandemic. After COVID-19 was imported into Africa, governments enforced anti-COVID-19 measures such as social distancing, quarantine, the wearing of masks and handwashing. Following the lifting-up of general population mandatory quarantine measures, the rate of compliance to those anti-COVID-19 measures decreased in Senegal (Mbow et al. 2020) and in other parts of Africa. Moreover, it was reported that the wearing of face masks in Africa is very controversial due to shortages in their stocks and uncertainty around the quality of masks (Aloui-Zarrouk et al. 2020).

In a paper, Mbow and co-workers speculated on possible reasons for the low COVID-19 incidence and severity in Africa (Mbow et al. 2020). They excluded the possibility of a more virulent SARS-CoV-2 strain in Africa, as discussed by other scientists (Grubaugh et al. 2020). The SARS-CoV-2 genomic data from Africa represent a small fraction of the total SARS-CoV-2 sequences from GISAID, and more sequences are needed to fully understand the molecular epidemiology and evolution of SARS-CoV-2 in Africa.

Several papers support the idea that youthfulness of the Africans may be associated with the more rapid development of protective immunity among the majority of Africans (Margolin et al. 2020; Njenga et al. 2020; Nordling 2020b). Indeed, Africa is the youngest continent with a median age of 19 years old (Desjardins 2019). Margolin and co-workers recently discussed the prospects for SARS-CoV-2 diagnostics, treatment and vaccines in Africa (Margolin et al. 2020). In their paper, the warm climate in Africa was considered as another possible reason for the low infection estimates in Africa because a winter seasonal pattern has been observed for other endemic human coronaviruses (Audi et al. 2020; Li et al. 2020b). If this claim is true, then the circulation of SARS-CoV-2 might also follow a certain seasonal trend, with a peak number of infections likely observed during winter. The epidemiological surveillance of SARS-CoV-2 has proven important to detect marked rise in COVID-19 cases in winter and new variant emerged in the UK, South Africa and Brazil (World Health Organization 2021a, b).

### COVID-19 risk factors in Africa

Numerous studies have examined the risk factors for COVID-19 mainly among infected persons (Caramelo et al. 2020; Notari and Torrieri 2020; Ouchetto and Bourhanbour 2020; Yanga et al. 2020). Those studies report that older individuals are more likely to develop severe forms of COVID-19 as 30–65-year-old adults account for 71.45% of infected persons, while children under 10-year-old account for 0.35% of cases (Pavel et al. 2020; Wu and McGoogan 2020). Since Africa is the youngest continent (Desjardins 2019), Africans may be less susceptible to the SARS-CoV-2 infection because of their youthfulness (Margolin et al. 2020; Njenga et al. 2020; Nordling 2020b).

Obesity is one of the important and modifiable risk factors for COVID-19 (Jordan and Adab 2020), because it may increase the risk of severe outcomes (Sattar et al. 2020). A pooled analysis of trends in adult body mass index in 200 countries from 1975 to 2014 showed that the least proportion of obese individuals are located on the African continent (NCD-Risk-Factor-Collaboration 2016), whereas the disproportionate COVID‐19 mortality in African Americans and other disadvantaged groups in the USA and UK may be associated with the high prevalence of obesity among those sub-populations. Non‐Hispanic black adults (i.e. African Americans), especially African-American women (56.9%), have the highest prevalence of obesity and severe obesity compared with other US races and Hispanic‐origin groups (Hales et al. 2017).

There are controversies regarding the association between HIV and COVID-19 severity in Africa. Increased severity of COVID-19 infection was observed among HIV positive South-Africans (Davies 2020), whereas, HIV prevalence had a protective effect among Nigerians (Hassan et al. 2020). More studies are therefore needed to understand the impact of co-infections on the COVID-19 disease outcome.

Similarly, protection against SARS-CoV-2 by BCG vaccination is also a matter for debate. In a multiple regression modelling study, a country-based association between COVID-19 mortality/million and the presence of universal BCG vaccination before 1980 was observed (Szigeti et al. 2020). On the other hand, Hensel et al. (2020) found that protection against SARS-CoV-2 by BCG vaccination was not supported by epidemiological analyses (Hensel et al. 2020). Moreover, studies related to the impact of BCG vaccine on COVID-19 susceptibility do not suggest that BCG strain used in Africa for vaccination induces heterologous anti-SARS-CoV-2 immunity. For example, Hamiel et al. and Chakafana et al. found no correlation between BCG vaccination and COVID-19 mortality rates in Africa (Hamiel et al. 2020; Chakafana et al. 2020).

Blood groups have also been associated with COVID-19 disease susceptibility and severity. A genome-wide analysis study recently reported a protective effect in blood group O as compared with other blood groups (Ellinghaus et al. 2020). In agreement with this finding, a meta-analysis showed that individuals with blood group A are at higher risk of COVID-19 infection, while those with blood group O are at lower risk (Pourali et al. 2020). The relationship between blood grouping and susceptibility to COVID-19 in Africa is still unclear. Genome-wide association study data collected from Africans are scarce, as well as public blood group database from different countries. The availability of such data would enable scientist to assess the eventual relationship between blood groups and the low mortality and morbidity in Africa in the near future. It would be interesting to analyse the blood group distribution by each country and by continents to uncover whether the blood group plays a role in the currently low reported COVID-19 cases in Africa.

Globally, high cardiovascular disease and hypertension prevalence (Rath et al. 2020; Yanga et al. 2020) were directly associated with high COVID-19 infection severity. In an observational study, Chakafana et al. noted that observations in North African countries are concordant with global findings, while discordant patterns in sub-Saharan Africa were observed, as there was no direct relationship between COVID-19 mortality rates and cardiovascular disease burden (Chakafana et al. 2020). They hypothesized that, following SARS-CoV-2 infection, there may be differences in COVID-19 pathophysiology in different African regions. The prevalence of hypertension is highest in Africa, compared to other continents (World Health Organization 2017). Even if we consider the variables such as the prevalence of HIV, hypertension and cardiovascular diseases, the highest COVID-19 death rate in Africa (844 deaths per 1 million) is still nearly 2 times lower than in the death rate in UK (1821 deaths per 1 million) or USA (1606 deaths per 1 million) (Worldometers 2021). Therefore, comorbidities are less likely to account for the observed lower COVID-19 prevalence, despite the paucity of knowledge. In this regard, laboratory testing rate, genetic factors or immunological factors should be considered to understand low COVID-19 infection estimates in Africa. The data on testing of COVID-19 in Africa are scarce, but available sources suggest a low testing rate of COVID-19 in Africa (Sterck 2013; Ourworldindata 2021) could also account for the low number of reported cases. Therefore, analysis of excess death in 2020 should be conducted in Africa to find out whether those deaths may be attributable to COVID-19.

### The role of herbal medicine as a primary health care solution in Africa

Traditional medicine, in contrast to conventional medicine, is based on knowledge, skills, theories, beliefs and experiences indigenous to different peoples and cultures (World Health Organization 2013). Since the pre-colonial era, plant-based herbal preparations and finished herbal products have been widely used in African traditional medicine (Abdullahi 2011). It is estimated that traditional practitioners manage at least 80% of the healthcare needs of rural inhabitants in East Africa (Judith et al. 2016; Ndetei 2007; World Health Organization 2013) and nearly 80% of people in Africa regularly seek their services for their primary health needs (World Health Organization et al. 1983). This shows that decades after historical independence in Africa, herbal medicine has remained very popular in Africa because it is natural, cheaper, geographically more accessible, acceptable, and effective (Bamidele et al. 2009; Mahomoodally 2013). In addition, majority of rural dwellers in Africa tend to seek conventional medical care only if they could not be cured through traditional pharmacopeia.

The use of antimalarial herbal treatments is particularly common in Africa because malaria is the main cause of mortality and morbidity in Africa (Iley 2006). Since early in vitro studies found that antimalarial drugs block SARS-CoV-2 infection at low-micromolar concentration, the use of antimalarial herbal preparations has been promoted as the treatment of COVID-19 in Africa. In spite of the contradictory data regarding hydroxychloroquine (Boulware et al. 2020; Gautret et al. 2020), it seems that countries that primarily use antimalarial drugs as COVID-19 treatment see a slower dynamic of daily deaths (Izoulet 2020). Although not scientifically proven yet, it is possible that the Africans infected with SARS-CoV-2 have been treated by naturopathic doctors, using plant extracts that contain antiviral compounds. Despite the therapeutic benefits shown in some medicinal plants, some plant constituents are potentially mutagenic, carcinogenic, toxic, and teratogenic (Akintonwa et al. 2009; Gadano et al. 2006). In addition, it is necessary to accelerate ongoing preclinical and clinical studies in order to validate the efficacy and safety of all plants, which may have therapeutic properties against COVID-19, especially antimalarial plants (Schlagenhauf et al. 2020). Further research involving socio-anthropologists, biochemists, and phyto-therapists are needed to clarify the therapeutic role played by plant-based anti-COVID-19 traditional treatments.

### Immunological factors

Pathogen richness (the number of kinds), prevalence (number of cases) and their consequences vary dramatically among continents, and the African continent is known to harbour a widest variety of pathogens in the world (Dunn et al. 2010). It is very likely that regular exposure to malaria and other infectious diseases could activate the immune system to fight novel pathogens, including SARS-CoV-2 (Ekhayemhe and Akujuru 2020; Nordling 2020c). The high pathogen richness in Africa implies an early and long-term exposure to pathogens, which might lead to early immune cell activation and eventual long-term protection against a wider range of pathogens (Von Mutius 2007; Yazdanbakhsh et al. 2002). In that sense, recent studies have shown that T cells reactive to SARS-CoV-2 epitopes have been identified in individuals exposed to other mild forms of coronaviruses, suggesting some protective heterologous immunity (Grifoni et al. 2020; Pinto et al. 2020). Therefore, it is possible that African have been exposed to other kinds of milder coronaviruses, but prior sero-epidemiological data in Africa are scanty and it is unclear whether Africans have residuals cross-neutralizing antibodies against SARS-CoV-2. Early seroprevalence data suggest that Spanish Kenyans have had a level of exposure to SARS-CoV-2 similar to that of the Spanish (Uyoga et al. 2020). Therefore, immunity may play an important role in the low morbidity and severity of COVID-19 in Africa.

### Genetic factors may confer protection against SARS-CoV-2 infection in Africans

Genetic factors are associated with numerous communicable and non-communicable diseases. There is increasing evidence that the major genetic risk factor for severe COVID-19 is inherited from Neanderthals (COVID-19 Host Genetics Initiative 2020; Ellinghaus et al. 2020; Zeberg and Pääbo 2020). To support this idea, Zeberg et al. showed that the major genetic risk to COVID-19 is conferred by a 50-kilobase pair genomic segment that was inherited from the Neanderthals. In their paper, they analysed the geographic distribution of the Neanderthal core haplotype conferring risk for severe COVID-19 and found that this genomic segment is carried by around 50% of people in south Asia and around 16% of people in Europe (Zeberg and Pääbo 2020).

Available studies suggest that African ancestry may be associated with higher immune protection against infection. In an experimental study, Nédélec et al. compared the differences in transcriptional response to infection among individuals of African and European ancestry. The findings suggested that African ancestry is associated with a stronger inflammatory response against infections (Nédélec et al. 2016; Quach et al. 2016). If African ancestry was the only genetic susceptibility factor to SARS-CoV-2 infection, then African Americans wouldn’t have incurred the highly observed COVID-19 burden in the USA (CDC 2020; Hamidian Jahromi and Hamidianjahromi 2020). Therefore, it is possible that other genetic factors such as mannose-binding lectin gene polymorphisms (Chen et al. 2019; Zhang et al. 2020), histo-blood group antigens (Cuéllar-Cruz 2021) and ACE2 level of expression are associated with susceptibility to severe acute respiratory syndrome coronavirus infections (Gyebi et al. 2020; Liu et al. 2020). Therefore, large-scale population studies are needed to ascertain the role of genetic factors in the susceptibility to SARS-Cov-2 and severity of COVID-19.

Recent studies reported the implication of angiotensin-converting enzyme 2 (ACE2) and the transmembrane serine protease 2 (TMPRSS2) in the priming the SARS-CoV-2 infection (Hoffmann et al. 2020). Analysis of expression dataset has revealed that ACE2 and TMPRSS2 level vary according to age (Bunyavanich et al. 2020; Pavel et al. 2020). Moreover, TMPRSS2 genetic polymorphisms may affect its expression in some ethnic groups, thus modifying the susceptibility to SARS-CoV-2 cellular entry (Piva et al. 2021). Measuring the level of ACE2, TMPRSS, and other susceptibility markers across population and ethnicity might clarify the relationship between genetic factors and COVID-19.

### *R*_0 _estimates of SARS-CoV-2 transmission in Africa might have been overestimated

T cells reactive to SARS-CoV-2 epitopes have been identified in individuals exposed to other “common cold” coronaviruses, suggesting some protective heterologous immunity (Grifoni et al. 2020), a concept that has also been proposed for cross-reactive antibodies induced in patients with SARS-CoV and MERS infections (Pinto et al. 2020). As mentioned earlier, older age is associated with COVID severity (Mallapaty 2020). The basic reproduction number or basic reproduction ratio or rate (*R*_0_) is an epidemiologic metric used to describe the contagiousness or measure the transmission potential of infectious agents (Dietz 1993). The basic reproductive number for COVID-19 based on SIR model does not assume that the population is susceptible and does not consider age as a confounding variable (Cooper et al. 2020). The estimated COVID-19 basic *R*_0_ of 2.2 (Li et al. 2020a) means that, on average, each infected person spreads the virus to two persons. As the authors note, until this number falls below 1.0, it is likely that the outbreak will continue to spread. So far, *R*_0_ estimates of COVID-19 were computed under the assumption that the whole population is equally susceptible to the viral infection. But previous results have shown that age and past exposure to homologous antigens (“common-cold” coronaviruses) are important factors that determine the outcome SARS-CoV-2 infection. Hilton et al. focused on the age-structured transmission within the population, using data from China to estimate age-dependent susceptibility and synthetic age-mixing matrices to inform the contact network. Hilton et al. found the lowest *R*_0_ estimates in Africa (Hilton and Keeling 2020). We argue that more accurate *R*_0_ estimates for SARS-CoV-2 infection should not only consider age, but it should also take into account level of past exposure to homologous antigens.

## Conclusion

**Fig. 1:**
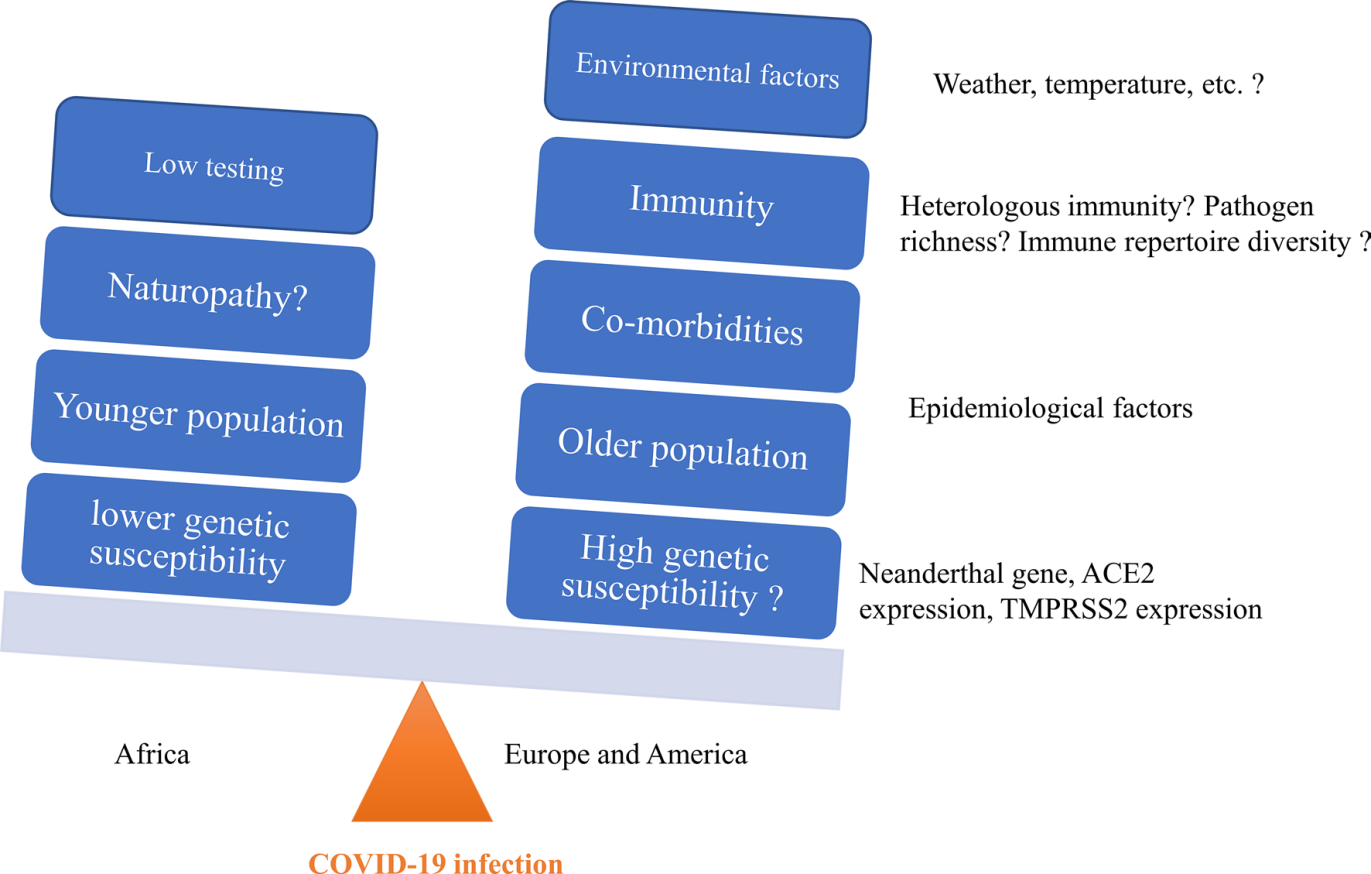
Summary of the reasons for the low burden of COVID-19 in Africa. The higher burden of COVID-19 in European and American continents and possibly responsible factors are shown

Given the current state of knowledge on COVID-19 and considering the results of previous articles, we have outlined different factors that may explain the apparent low rate of infection and death due to COVID-19 in Africa. As the pandemic continues, it is unknown whether the hotspot will move to the African continent. The differences in the incidence of COVID-19 are certainly related to multiple variables. In addition to the age, weather, comorbidities, genetic, immunological and socio-demographical, and even anthropological factors may be helpful to understand Africa’s impressively lower burden of COVID-19 (Fig. 1). However, newly emerged SARS-CoV-2 variants might confer different antigenicity to the virus and thus change the overall distribution of the observed number of COVID-19 cases. Active genomic surveillance and efficient testing would be of huge importance to control the spread of the novel coronavirus.

## Data Availability

Not applicable.

## Abbreviations

COVID-19: Coronavirus disease (COVID-19) is an infectious disease caused by a newly discovered coronavirus
SARS-CoV-2: Severe acute respiratory syndrome coronavirus 2 (SARS-CoV-2)
BCG: Bacille Calmette–Guérin
ACE2: Angiotensin-converting enzyme 2 (ACE2)
HLA: Human leukocyte antigen
GISAID: A global science initiative established in 2008 that promotes and provides open access to genomic data of influenza viruses and the SARS-CoV-2
HIV: Human immunodeficiency virus
FDA: United States Food and Drug Administration
TMPRSS2: Transmembrane protease, serine 2
SARS-CoV: Severe acute respiratory syndrome coronavirus 1
MERS-CoV: Middle East respiratory syndrome coronavirus
